# Carrier frequency and incidence estimation of Smith–Lemli–Opitz syndrome in East Asian populations by Genome Aggregation Database (gnomAD) based analysis

**DOI:** 10.1186/s13023-021-01789-2

**Published:** 2021-04-09

**Authors:** Jong Eun Park, Taeheon Lee, Kyeongsu Ha, Chang-Seok Ki

**Affiliations:** 1 Department of Laboratory Medicine, Hanyang University Guri Hospital, Hanyang University College of Medicine, Guri, Republic of Korea; 2 Green Cross Genome, 107, Ihyeon-ro 30beon-gil, Giheung-gu, Yongin-si, Gyeonggi-do 16924 Republic of Korea

**Keywords:** Smith–Lemli–Opitz syndrome, *DHCR7*, gnomAD, Carrier frequency, East asian

## Abstract

**Background:**

Smith–Lemli–Opitz syndrome (SLOS) is an autosomal, recessively inherited congenital malformation syndrome characterized by multiple congenital anomalies such as microcephaly with mental defects, distinctive facial features, genital abnormalities, and 2–3 syndactyly of the toes. SLOS is caused by defective 7-dehydrocholesterol reductase, which is encoded by the *DHCR7* gene. This study aimed to analyze the carrier frequency and expected incidence of SLOS in East Asians and Koreans using exome data from the Genome Aggregation Database (gnomAD) through the 2015 American College of Medical Genetics and Genomics and the Association for Molecular Pathology guideline (2015 ACMG-AMP guideline).

**Methods:**

We analyzed 9197 exomes for East Asian populations from gnomAD, comprising 1909 Korean, 76 Japanese, and 7212 other East Asian populations. All identified variants were classified according to the 2015 ACMG-AMP guideline.

**Results:**

According to the 2015 ACMG-AMP guideline, 15 pathogenic variant/likely pathogenic variant (PV/LPV) cases were identified in 33 East Asian individuals (33/9191 = 0.4%). Among them, four PVs/LPVs were identified in 19 Korean individuals (19/1909 = 1.0%). The predicted incidence, based upon the carrier rates of PV/LPV of *DHCR7* alleles, is 1 in 310,688 in East Asians and l in 40,380 in Koreans.

**Conclusions:**

This study is the first to identify carrier frequencies in East Asians and Koreans using gnomAD. It was confirmed that East Asians (0.4%) had a lower carrier frequency than did other ethnicities (1–3%) and Koreans (1.0%) had similar or lower carrier frequencies than other ethnicities. The variant spectrums of *DHCR7* in East Asian and Korean populations differed greatly from those of other ethnic groups.

## Background

Smith–Lemli–Opitz syndrome (SLOS, OMIM #270,400) is an autosomal, recessively inherited congenital malformation syndrome, which is an inborn error of cholesterol metabolism [1]. SLOS was first described in 1964 in three unrelated children, which showed a similar pattern of multiple congenital anomalies such as microcephaly with mental defects, distinctive facial features, genital abnormalities, and 2–3 syndactyly of the toes [2]. SLOS is caused by a functional defect in 7-dehydrocholesterol reductase (DHCR7), which is encoded by the *DHCR7* gene. DHCR7 catalyzes the conversion of 7-dehydrocholesterol (7-DHC) to cholesterol, which is the last step in cholesterol biosynthesis [3]. The genetic loss of DHCR7 enzyme activity leads to reduced cholesterol synthesis, with accumulation the precursor product, 7-DHC (as well as 8-DHC, its spontaneous isomer).

SLOS incidence has been reported to be between 1/10,000 and 1/70,000 in populations, primarily studied in Caucasians, while its incidence in East Asians is not well known [4, 5]. Carrier frequency estimates depend on the variant evaluated and the population studied. Based on the c.964-1G > C variant, the most common pathogenic variant in SLOS, the carrier frequency is known to be approximately 1% in Caucasians, but up to 3% has been reported [6–8]. Carrier frequency studies are extremely rare in the East Asian population. One such study was carried out using large-scale data, including 1000 Genomes phase 1 with 286 East Asians, and the carrier frequency of East Asians was reported to be 1.6% [9]. Moreover, the cumulative carrier frequency of 13,546 East Asians, who performed elected expanded carrier screening, was 0.1% [10].

Recent studies on the genetic cause of stillbirth have reported associations with SLOS [11]. A difference exists between the expected incidence of SLOS and its actual incidence; this is estimated to be different as approximately 42–88% of affected conceptuses experience prenatal death [10]. Moreover, exposure to drugs such as aripiprazole or trazodone increases 7-DHC concentration, thus increasing the vulnerability of the *DHCR7* variant carrier; and this is estimated to affect fetal development during pregnancy [12, 13]. Therefore, it is important to know the carrier frequency of pathogenic variant (PV)/likely pathogenic variant (LPV) *DHCR7* alleles.

The Genome Aggregation Database (gnomAD) is a popular genomic database used worldwide, and gnomAD V2 is composed of 125,748 exomes and 4,359 genomes [14]. gnomAD V2 contains exome data collected from 9197 East Asians, including 1909 Koreans, and is suitable for East Asian studies as it contains the largest amount of East Asian data among the genome databases released to the public. We also interpreted the *DHCR7* variants according to the 2015 American College of Medical Genetics (ACMG) and Genomics and the Association for Molecular Pathology (AMP) guidelines, which have been widely adopted in clinical practice [15]. This study aimed to analyze the carrier frequency and expected incidence of SLOS in East Asians and Koreans using exome data from the 2015 ACMG-AMP guidelines.

## Methods

### gnomAD East Asian population data

gnomAD data (v2.1.1) for the *DHCR7* gene were obtained from https://gnomad.broadinstitute.org/. We analyzed 9197 East Asian exomes, of which 1909 were from Koreans, 76 were from Japanese, and 7212 were from other East Asian populations. Variants predicted to have a large impact on protein function, including missense, nonsense, frameshifts, in-frame insertions/deletions variants, or changes affecting the consensus splice site sequences, were filtered.

### ***DHCR7*** variant classification and statistical analysis

Filtered variants were interpreted using the 2015 ACMG-AMP guideline. This guideline recommends the classification of variants into five categories: PV, LPV, variant of uncertain significance, likely benign variant, and benign variant. Various in silico tools such as SIFT (http://sift.jcvi.org), PolyPhen-2 (http://genetics.bwh.harvard.edu/pph2), CADD (http://cadd.gs.washington.edu), and GERP (http://mendel.stanford.edu/sidowlab/downloads/gerp/index.html) were used. All *DHCR7* variants identified in gnomAD were additionally classified according to the Human Gene Mutation Database (HGMD) and ClinVar. The HGMD professional database (http://www.hgmd.org/, release 2020.4) is a comprehensive collection of germline variants, categorized into six categories. ClinVar (https://www.ncbi.nlm.nih.gov/clinvar/) is a freely available archive that provides classification of variants interpreted by clinical laboratories.

### SLOS carrier frequency and incidence estimation

East Asian and Korean carrier frequencies were calculated for the *DHCR7* gene using gnomAD. We used those classified as the PV and LPV according to the 2015 ACMG-AMP guideline interpretation, the disease-causing variant (DM) in HGMD, and those classified as PV and LPV in ClinVar for carrier frequency analysis. Thereafter, we estimated the incidence of SLOS based on that carrier frequency of PV/LPV *DHCR7* alleles and the Hardy–Weinberg equilibrium principle (1 = *p*^2^ + 2*pq* + *q*^2^). The major allele is *p* (non-disease), the minor allele is *q* (disease). The major allele *p* is assumed to be approximately 1. 2*pq* and represents the carrier, and *q*^2^ represents the disease. By calculating the *q* value based on the carrier frequency obtained from gnomAD, the estimated disease incidence *q*^2^ was predicted. MedCalc ver. 11.5.1.0 (MedCalc Software, Maiakerke, Belgium) was used for statistical analysis, and 95% confidence intervals (CIs) were calculated for each value.

## Results

In 9197 East Asian exomes there were 61 *DHCR7* gene variants, of which 57 were missense, two were nonsense, one was stop-lost variant, and one was in-frame deletion variant. In 1909 Korean exomes there were 16 variants, of which 15 were missense and one was nonsense. These variants were classified according to the 2015 ACMG-AMP guideline and two disease classification databases, HGMD and ClinVar (Table 1).

**Table 1:** Carrier frequency and estimated incidence of Smith–Lemli–Opitz syndrome in East Asian and Korean

	Variants (n)	Total individuals (n)	Carrier frequency (%), (95% CI)	Estimated incidence (1/n), (95% CI)
gnomAD East Asian exomes (n = 9197)				
2015 ACMG-AMP (PV/LPV)	15	33	0.4 (0.2–0.5)	1/310,688 (1/157,533–1/655,641)
HGMD (DM)	9	21	0.2 (0.1–0.3)	1/767,209 (1/328,405–1/2,003,437)
ClinVar (PV/LPV)	10	23	0.3 (0.2–0.4)	1/639,583 (1/284,141–1,592,214)
gnomAD Korean exomes (n = 1909)				
2015 ACMG-AMP (PV/LPV)	4	19	1.0 (0.6–1.6)	1/40,380 (1/16,557–1/111,408)
HGMD (DM)	3	12	0.6 (0.3–1.1)	1/101,230 (1/33,178–1/379,165)
ClinVar (PV/LPV)	2	11	0.6 (0.3–1.0)	1/120,472 (1/37,631–1/483,595)

2015 ACMG-AMP, 2015 American College of Medical Genetics and Genomics and the Association for Molecular Pathology guideline; 95% CI, 95% confidence intervals; DM, disease-causing variant; gnomAD, Genome Aggregation Database; LPV, likely pathogenic variant; PV, pathogenic variant

According to the 2015 ACMG-AMP guideline, 15 PV/LPV cases were identified in 33 East Asian individuals (33/9191 = 0.4%). Among them, 19 Koreans were identified as four kinds of PVs/LPVs (19/1909 = 1.0%). The estimated incidence of SLOS was 1 in 310,688 in East Asians and l in 40,380 in Koreans. Based on HGMD, the carrier frequency was 0.2% in East Asians and 0.6% in Koreans. Estimated incidences were 1 in 767,209 in East Asians and l in 101,230 in Koreans. Based on ClinVar, the carrier frequency was 0.3% in East Asians and 0.6% in Koreans. Estimated incidences were 1 in 639,583 in East Asians and l in 120,472 in Koreas.

*DHCR7* PVs/LPVs found in East Asians and Koreans are summarized in Table 2. The c.907G > A (p.Gly303Arg) variant was most common in Koreans, and although this was the most common variant found in East Asia in our study, it was only identified in Koreans. When comparing the PVs/LPVs found in East Asians and Koreans with other ethnicities, PVs/LPVs identified in East Asians and Koreans were not found in Ashkenazi Jewish, European (Finnish), and Latino populations, except for the c.907G > A variant. The c.964-1G > C variant, the most commonly known pathogenic variant in SLOS, was not identified in East Asians and Koreans.

**Table 2:** Pathogenic variants and likely pathogenic variants of East Asian and Korean in gnomAD population

Nucleotide change	Amino acid change	gnomAD allele frequency (allele count/allele number)								
		Korean (n = 1909)	East Asain (n = 9197)	African (n = 8128)	Latino (n = 17,296)	Ashkenazi Jewish (n = 5040)	European (Finnish) (n = 10,824)	European (non-Finnish) (n = 56,885)	Other (n = 3070)	South Asian (n = 15,308)
c.16 C > T	p.Gln6Ter	0	5.437E−05 (1/18,394)	0	0	0	0	0	0	0
c.356 A > G	p.His119Arg	0	5.44E−05 (1/18,382)	0	0	0	0	7.046E−05 (8/113,532)	0	0.00026228 (8/30,502)
c.724 C > T	p.Arg242Cys	0	5.437E−05 (1/18,394)	0.0001847 (3/16,244)	0	0	0	0.0001672 (19/113,650)	0.0001630 (1/6,136)	3.2663E−05 (1/30,616)
c.725G > A	p.Arg242His	0	5.437E−05 (1/18,394)	0	0	0	0	3.52E−05 (4/113,650)	0	0
c.730G > A	p.Gly244Arg	0	5.437E−05 (1/18,392)	0	0	0	0	8.8E−06 (1/113,638)	0	0.0001960 (6/30,616)
c.852 C > A	p.Phe284Leu	0	5.446E−05 (1/18,362)	0	0	0	0	0	0	0
c.860 A > G	p.Asn287Ser	0	5.444E−05 (1/18,370)	0	0	0	0	0	0	0
c.907G > A	p.Gly303Arg	0.0026192 (10/3,818)	0.0005439 (10/18,386)	0.0002461 (4/16,254)	2.892E-05 (1/34,580)	0	4.634E-05 (1/21,578)	6.156E−05 (7/113,704)	0	0
c.1055G > A	p.Arg352Gln	0.0002622 (1/3,814)	5.473E−05 (1/18,270)	0.0001248 (2/16,022)	0	0	0	1.786E−05 (2/111,962)	0	3.2686E−05 (1/30,594)
c.1084 C > A	p.Arg362Ser	0	5.462E-05 (1/18,308)	0	0	0	0	0	0	0
c.1085G > A	p.Arg362His	0	5.46E−05 (1/18,316)	0	0	0	0	4.475E−05 (5/111,722)	0	9.8026E−05 (3/30,604)
c.1139G > T	p.Cys380Phe	0.001048 (4/3,816)	0.0002188 (4/18,280)	0	0	0	0	0	0	0
c.1140 C > A	p.Cys380Ter	0.001048 (4/3,818)	0.0002187 (4/18,286)	0	0	0	0	0	0	0
c.1190 C > T	p.Ser397Leu	0	5.541E−05 (1/18,046)	0	0	0	0	1.833E−05 (2/109,128)	0	0
c.1426T > C	p.Ter476GlnextTer51	0	0.0002189 (4/18,276)	0	0	0	0	0	0	0

gnomAD, Genome Aggregation Database

## Discussion

In this study, the carrier frequency of PV/LPV *DHCR7* alleles and estimated incidence of SLOS were analyzed for East Asians and Koreans using gnomAD. The carrier frequency of East Asians was 0.4%, which was lower than that of other ethnic groups (1–3%) [6, 7]. Among East Asians, the carrier frequency of Koreans was 1.0%, which was similar to or lower than that of other ethnicities (1–3%). Based on disease classification databases, HGMD and ClinVar, carrier frequencies were 0.2–0.3% in East Asians and 0.6% in Koreans. Compared with previous studies on East Asians, carrier frequency in gnomAD is located between the previous reports (0.1 to 1.6%) [9, 10].

The carrier frequency is thought to differ between studies because of differences in variant interpretation and analysis method. In case of Cross et al.’s study, the entire *DHCR7* region was analyzed in carrier frequency analysis using 1000 Genomes phase 1, including 286 East Asian genomes, and the criteria for the pathogenic variant were whether it was a previously reported variant and whether it was ‘Probably Damaging’ or ‘Possibly Damaging’ in Polyphen-2 [9]. As the 2015 ACMG-AMP guideline is more stringent in variant classification, a previous study has shown a relatively higher carrier frequency than this. In another study, 13,546 East Asians were analyzed, of which 3102 East Asians were analyzed using next-generation sequencing (NGS), and another 10,444 were analyzed using targeted genotyping that could only identify 13 major *DHCR7* variants [10]. The carrier detection rate according to the method difference was different for each ethnic group. In Ashkenazi Jewish or Africa, compared to NGS, 100% of the variants were identified in targeted genotyping, while East Asians were found to miss 80% when only performing targeted genotyping. In fact, the cumulative East Asian carrier frequency confirmed by Lazarin et al.’s study was 0.10%. Each carrier frequency was 0.16% via NGS and 0.08% via targeted genotyping. Therefore, the East Asian carrier frequency would have been underestimated by the targeted genotyping method. In this study, 9197 East Asian genomes, more than in previous studies, were analyzed for variants in the entire *DHCR7* gene region using the 2015 ACMG-AMP guideline. It is thought that a more accurate carrier frequency was reflected.

The PV/LPV identified in this study was found to have a completely different variant spectrum pattern from other ethnicities. c.964-1G > C, p.Thr93Met, p.Trp151Ter, p.Val326Leu, p.Arg404Cys, and p.Arg352Trp variants, known to be frequently reported in SLOS patients, were not found in East Asians in the gnomAD [16]. Conversely, PV/LPV identified in East Asians and Koreans were not found in Ashkenazi Jews, Europeans (Finland), and Latinos, except for the c.907G > A variant. From this, it could be inferred that the variant spectrum of the *DHCR7* gene differs between East Asian races and other ethnicities.

In particular, c.964-1G > C is the most common variant in Caucasians, and several studies on carrier frequency and estimated incidence using the c.964-1G > C variant have been reported [6]. To the best of our knowledge, c.964-1G > C mutations have not been reported in SLOS patients in three East Asian countries (Korea, China, and Japan). According to Cross et al., a Han Chinese individual in Beijing was confirmed to have a c.964-1G > C variant allele in 1000 Genomes phase 1 [9]. In 1000 Genomes phase 3, and in 504 East Asians, c.964-1G > C was not found [17]. According to Lazarin et al., two c.964-1G > C alleles were identified in 13,546 East Asians by targeted genotyping [10]. Considering that the c.964-1G > C variant was not found in this study, it is thought that the c.964-1G > C mutation is rarely found in East Asia.

In East Asians and Koreans, the c.907G > A (10/18,386 alleles in East Asians, 10/3,818 alleles in Koreans) variant was most frequently identified. The c.907G > A variant has been primarily reported in the Korean and Japanese populations [18, 19]. In Korean SLOS patients, the c.1054 C > T (p.Arg352Trp)(7/16 alleles) variant is most common, followed by c.907G > A (4/16 alleles) [20]. The c.1055G > A (p.Arg352Gln) (9/13 alleles) variant is most common in Japanese SLOS patients, followed by the c.907G > A variant (2/13 alleles) [18].

SLOS incidence in Caucasians is known, while its incidence in East Asians was unknown. When calculated by the Hardy-Weinberg equation, the incidence in East Asians is predicted to be 1/310,688 and 1/40,380 in Koreans. The incidence of SLOS in East Asians was predicted to be lower than that in other ethnicities, and lower or similar in Koreans. Considering the still birth rate, the actual incidence of SLOS in East Asians and Koreans may be lower than that which was estimated by SLOS carrier frequency.

According to data from the Korean Statistical Information Service (http://kosis.kr/; accessed on 02 November 2020) in 2019, the total population of Korea was 51.8 million with 302,676 births. Based on the carrier frequency of PV/LPV *DHCR7* alleles in this study, the number of carriers is estimated to be 0.52 million in total, and 3,027 in newborns per year. The estimated incidence of SLOS in Korea based on the Hardy–Weinberg equilibrium is seven cases per year. However, considering the utero mortality rate, it is predicted that the actual number of patients born with SLOS will be < 7.

Recent studies on the genetic cause of stillbirth have reported associations with SLOS [11]. There is a difference between the expected incidence of SLOS and its actual incidence; this difference is possibly because approximately 42–88% of affected conceptuses experience prenatal death [10]. In addition, exposure to drugs such as aripiprazole or trazodone increases the 7-DHC concentration in the *DHCR7* mutation carrier making it vulnerable, and is estimated to affect fetal development during pregnancy [12]. Therefore, it is important to know the carrier frequency of PV/LPV *DHCR7* alleles.

Recently, studies have been reported on the risk of aripiprazole, which is frequently used in the treatment of schizophrenia or bipolar disorder, in a *DHCR7* variant carrier [12, 13]. Aripiprazole increases the 7-DHC concentration, such that the *DHCR7* variant carrier is vulnerable to the drug. Aripiprazole is a known drug, often used in pregnant women. Animal studies have shown that *DHCR7* carriers affect fetal brain development when aripiprazole is administered during pregnancy [13]. In addition to aripiprazole, trazodone, or haloperidol, which can increase 7-DHC concentration, might not be safe in *DHCR7* variant carriers [13]. In this study, 0.4% of East Asians and 1.0% of Koreans were identified as *DHCR7* PV/LPV carriers. Among the *DHCR7* variant carriers, attention should be paid to drug use, especially in pregnant women.

This study has some limitations. We did not analyze structural variations, including the large deletion/insertion of the *DHCR7* gene. According to Lanthaler et al., in two of the 12 SLOS patients, where only one variant was identified, a large deletion of the *DHCR7* gene was confirmed through multiplex ligation-dependent probe amplification [21]. Nonetheless, this study makes several valuable contributions. This is the largest study among those performed in East Asia that analyzed the entire *DHCR7* gene. To the best of our knowledge, there have been no large-scale population studies of carrier frequencies and estimated SLOS incidence in Koreans. We believe that this study more accurately predicted the carrier frequency of SLOS in East Asia and Korea.

## Conclusions

This study is the first to identify carrier frequencies in East Asians and Koreans using gnomAD. We confirmed that East Asians had a lower carrier frequency than other ethnicities, and Koreans had lower or similar carrier frequencies compared to other ethnicities. The variant spectrum of *DHCR7* in East Asian and Korean populations differed greatly from those of other ethnic groups. Our data are expected to serve as a reference for further investigation of SLOS in the East Asian and Korean population.

## Data Availability

All data are available by corresponding author upon reasonable request.

## Abbreviations

2015 ACMG-AMP guideline: 2015 American College of Medical Genetics and Genomics and the Association for Molecular Pathology guideline
7-DHC: 7-dehydrocholesterol
DHCR7: 7-dehydrocholesterol reductase
ACMG: American College of Medical Genetics
AMP: Association for Molecular Pathology
CIs: Confidence intervals
DM: Disease-causing variant
gnomAD: Genome Aggregation Database
HGMD: Human Gene Mutation Database
LPV: Likely pathogenic variant
NGS: Next-generation sequencing
PV: Pathogenic variant
SLOS: Smith–Lemli–Opitz syndrome
